# Quantification of intervertebral disc degeneration using dual-energy computed tomography collagen maps: a comparative study with MRI Pfirrmann grading

**DOI:** 10.1038/s41598-026-69070-9

**Published:** 2026-09-03

**Authors:** Lara Farkic, Carsten Stelbrink, Friedemann Goehler, Kerstin Rubarth, Yan Klosterkemper, Friederike Schömig, Matthias Pumberger, Denis Poddubnyy, Dominik Deppe, Robert Biesen, Mikhail Protopopov, Torsten Diekhoff, Julian Pohlan

**Affiliations:** 1 Department of Radiology, Charité-Universitätsmedizin Berlin, Campus Mitte, Humboldt-Universität zu Berlin, Freie Universität Berlin, 10117 Berlin, Germany; 2 Institute for Biometry and Clinical Epidemiology, Charité-Universitätsmedizin Berlin, Campus Mitte, Humboldt-Universität zu Berlin, Freie Universität Berlin, 10117 Berlin, Germany; 3 Institute of Medical Informatics, Charité-Universitätsmedizin Berlin, Campus Mitte, Humboldt-Universität zu Berlin, Freie Universität Berlin, 10117 Berlin, Germany; 4 Center for Musculoskeletal Surgery, Charité - Universitätsmedizin Berlin, Campus Mitte, Humboldt-Universität zu Berlin, Freie Universität Berlin, Campus Mitte, 10117 Berlin, Germany; 5 Department of Gastroenterology, Infectiology and Rheumatology (including Nutrition Medicine), Charité-Universitätsmedizin Berlin, Campus Benjamin Franklin, Humboldt-Universität zu Berlin, Freie Universität Berlin, 12203 Berlin, Germany; 6 Department of Rheumatology and Clinical Immunology, Charité-Universitätsmedizin Berlin, Campus Mitte, Humboldt-Universität zu Berlin, Freie Universität Berlin, 10117 Berlin, Germany; 7 UCB Pharma GmbH , Rolf-Schwarze-Schütte-Platz 1, 40789 Monheim am Rhein, Deutschland; 8 Department of Radiology, Immanuel Klinik Rüdersdorf, Universitätsklinikum der Medizinische Hochschule Brandenburg, Seebad 82/83, 15562 Rüdersdorf, Germany; 9 Fliedner University of Applied Sciences, Geschwister-Aufricht-Straße 9, 40489 Düsseldorf, Germany; 10 Departement of Trauma and Orthopedic Surgery, Protestant Hospital of BethelFoundation, University OWL of Bielefeld University, Bielfeld, Germany

**Keywords:** DECT cMaps, Intervertebral disc degeneration, Pfirrmann-grade, Diseases, Health care, Medical research

## Abstract

To determine the correlation between quantitative HU values derived from DECT collagen maps and the MRI-based Pfirrmann grade. We retrospectively reviewed IVDs in patients who underwent both spinal DECT and MRI within one year. CT scans were acquired using single-source DECT at 80 and 135-kVp with automatic exposure control. Three readers scored IVDs according to Pfirrmann et al. on T2-weighted MRI images. Quantitative evaluation involved placing three standardized regions of interest in each IVD in DECT-based cMaps; in corresponding 135-kVp DECT scans and MRI images. Mean density measurements of AF and NP were compared with PF-grades using linear mixed model analysis. We included 339 IVDs of 97 patients [mean age 59.1 years]; (53 women [mean age 63.5 years] and 44 men [mean age 53.8 years]). cMap analysis revealed a significant difference of AF and NP collagen and chondroitin density between PF-grade I and V(AF: MD = − 31.1HU (95% CI − 59.5 to − 2.7), *p* = 0.032;NP:MD = − 37.8HU (95% CI − 73.6 to − 2.0), *p* = 0.038). 135-kVp DECT showed no IVD density reduction corresponding to PF-grades. MRI showed significant reduction of signal intensities in AF and NP with increasing PF-grades. IVD collagen and chondroitin density declined with increasing PF-grade in DECT-based cMaps, suggesting DECT-based cMaps as an additional tool for IVD classification.

**Supplementary Information:**

The online version contains supplementary material available at https://doi.org/10.1038/s41598-026-69070-9.

## Introduction

MRI is currently considered the imaging standard of reference for the diagnostic assessment of intervertebral disc (IVD) pathologies such as intervertebral disc degeneration (IDD). Pfirrmann et al. introduced the commonly applied morphologic grading system for MRI, attained from T2-weighted images, based on the following criteria: MRI signal intensity (SI), disc structure and height as well as distinction between nucleus pulposus (NP) and annulus fibrosus (AF)^1^. Nevertheless, the diagnostic accuracy of morphologic grading may be degraded by effects of variable imaging conditions. Furthermore, MRI has clinical contraindications and practical limitations, including limited availability, long acquisition times and patient factors such as claustrophobia or the presence of cardiac devices and magnetic implants^2^.

CT is primarily used to assess the bone structure of vertebral bodies and facet joints. Nevertheless, its visualization of soft tissue structures is limited by contrast resolution, which is why newer techniques such as DECT have been increasingly studied in the analysis of spinal pathologies^3^.

The spectrum of DECT post-processing techniques applied to spinal imaging has expanded considerably, demonstrating remarkable versatility in evaluating various aspects of spinal pathology. These include virtual non-calcium (VNCa) imaging for enhanced visualization of disc herniation and characterization of lumbar disc degeneration^4,5^, and electron density (ED) mapping for improved detection of nerve root impingement^6^. The VNCa DECT postprocessing algorithm provides indirect measurements of collagen and chondroitin content by virtually subtracting calcium from the image, which is usually not present in the disc. Therefore, quantitative measurements on these post-processed images should not be different from standard CT when it comes to depicting the AF and the NP.

Among DECT post-processing algorithms, collagen mapping (cMap) represents a targeted imaging approach that aligns with IDD pathogenesis. As the disc degeneration progress involves extracellular matrix breakdown- including proteoglycan loss, reduced hydration, and collagen network disruption—cMaps offer a targeted assessment tool that addresses these structural alterations^7–10^.

The Application of the DECT-collagen material decomposition algorithm allows visualization and differentiation of collagenous tissues within various anatomical structures^11^, considered to reflect a specific sensitivity to the discs main structural components collagen and chondroitin sulphate^12^ , which are thought to play a role in the development of IDD^9,13^. This DECT postprocessing algorithm has already proven useful for the imaging of other collagen-rich musculoskeletal tissues like ligaments and tendons^14–16^. Recent studies successfully revealed the feasibility of using colour-coded DECT collagen mapping as an additional tool for the morphological assessment of thoracic and lumbar disc degeneration^17,18^. However, while these studies established the qualitative potential of collagen maps for disc classification, quantitative potential has not yet been assessed. Given DECT's potential as a useful additional tool for characterizing collagenous structures and associated osseous pathology, we therefore conducted this study to investigate whether quantitative DECT can determine the severity of IDD based on the imaging reference standard, i.e., MRI. The primary objective was to determine the correlation between quantitative HU values derived from DECT collagen maps and the MRI-based Pfirrmann grade.

## Materials and methods

### Ethics approval and informed consent

This study included patients who were participants of earlier studies conducted at our department. Included studies were approved by the local ethics committee Charité—Universitätsmedizin Berlin IRB under (EA1/230/19,EA1/011/20,EA1/372/14, EA1/086/16). The requirement for patient informed consent was waived by the local ethics committee Charité—Universitätsmedizin Berlin IRB due to the retrospective nature of the study, which involved de-identified routine clinical data. We confirm that all research was performed in accordance with applicable ethical standards and relevant guidelines.

### Patient selection

To identify patients for this retrospective clinical analysis, we searched the radiology database for patients who underwent both clinically indicated DECT and (1.5 or 3.0T) MRI within one year from March 2015 to July 2021. All computed tomography scans were acquired using single-source DECT at 80 and 135kVp with automatic exposure control. The maximum interval of one year between DECT and MRI was chosen pragmatically to obtain an adequate sample size from routinely acquired clinical examinations and were defined before data analysis. The observed intervals were considerably shorter than this maximum (see Results). All included IVDs were evaluated for interval/intermodal changes between the examinations, and no traumatic events involving the included spinal segments were documented in the available clinical records within the examination times.

The 97 subjects included in the present analysis were drawn from four earlier studies conducted at our department^19–22^, which used the same routinely acquired imaging data to address different research questions (inflammatory, traumatic and morphological) (see Supplementary material 10).

### Disc selection

**Fig. 1:**
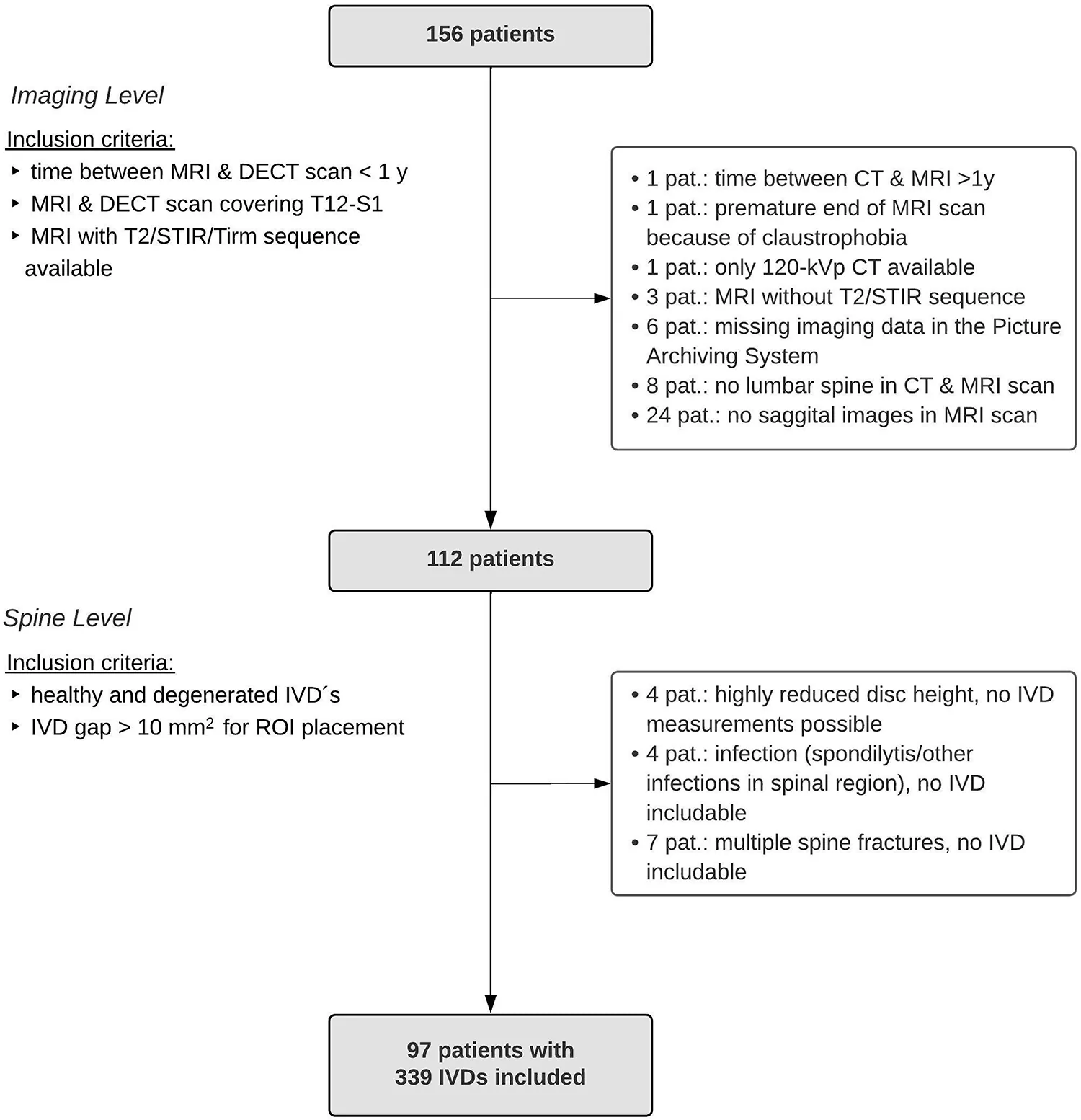
Flow chart of patient selection. We retrospectively included patients from separate studies. We searched for patients who underwent DECT and MRI within a timeframe of one year. Of 156 potentially eligible patients, a total of 97 patients with 339 IVDs were included. DECT, dual-energy computed tomography; MRI, magnetic resonance imaging; IVD, intervertebral disc

Normal and degenerative IVDs from the thoracic to the sacral region (T12 through S1) were considered for inclusion. Exclusion criteria were incomplete MRI/CT imaging data, known inflammation or infection of the discs and vertebral bodies (discitis/spondylodiscitis) and discs adjacent to an acutely fractured vertebral body or in an area with an earlier surgical intervention like spondylodesis with implant placement. In case of infection, two IVD compartments, one above and one below the infected disc or vertebral body, were excluded from analysis (Fig. 1).

### MRI rating

MRI protocols were not standardized, as both images performed in our clinic as well as externally obtained images were used. We included T2 weighted, short-tau inversion recovery (STIR), turbo inversion recovery magnitude (TIRM), and T2 turbo-spin echo (TSE) images conducted from various 1.5 and 3T MRI scanners in our analysis. Available slice thicknesses ranged from 3.0 to 5.0 mm. For the reading sessions, all included IVDs were cropped from the original image, leaving just the IVD with the adjacent vertebral bodies, allowing the isolated assessment of the IVD and minimalizing possible bias from more degenerated IVDs. Three readers, blinded to clinical and MRI findings, independently rated midsagittal images. Reader 1 had 15 years of imaging experience (radiologist specializing in musculoskeletal diseases) while readers 2 and 3 were radiologists with five years of imaging experience. Information on the IVD level and MRI sequence used was available to the readers. The readers were asked to assign one of the five Pfirrmann grades to each included IVD present. The final PF-grade of a disc was defined as the grade by at least two out of three readers. In case of disagreement between all readers, a consensus rating with all three observers was performed.

### DECT imaging and reconstruction

DECT imaging was performed on a 320-row single-source CT scanner (Aquilion ONE Vision, Canon Medical Systems) following the standard institutional dual-energy protocol, using sequential volume acquisition at 80 and 135-kVp with a rotation time of 0.275 s (0.5 s changeover). Automatic exposure control was applied for both energy levels. Primary dual-energy datasets were generated with a slice thickness of 0.5 mm from the raw data using iterative reconstruction (AIDR-3D, standard level) and a medium soft-tissue kernel without beam hardening compensation. cMaps were reconstructed at the CT console using a raw-data-based three-material decomposition algorithm (Dual-Energy Raw Data Analysis, version 6.0) with an established gradient of 1.10. Images were reconstructed in black and white in the sagittal plane with 3.0 mm slice thickness. The examinations had a mean DLP of 558.0 (SD 342.2, range 60.5–1605.6) mGy*cm and a mean CTDIvol of 14.8 (SD 10.1, range 1.8–53.2) mGy. Scan coverage varied depending on the clinical indication.

All measurements and image grading were performed at a workstation with a high-resolution monitor using Horos (version 3.3.6, Horos project, based onOsiriX™ open source).

### Quantitative analysis

Quantitative measurements of CT attenuation in Hounsfield units (HU) were obtained using three elliptical regions of interest (ROIs, each > 10 mm^2^) per disc, placed on midsagittal images in the anterior anulus fibrosus (AF), the centre of the nucleus pulposus (NP) and the posterior AF, in both the 135-kVp DECT images and the cMaps (Supplementary Fig. 4). ROI sizes varied according to the size of the disc but did not fall below the minimum of 10mm^2^. Representative ROI measurements were performed to ensure precise ROI placement, excluding bony structures of the adjacent vertebral bodies, additionally considering the impact of partial volume effects. Slice thicknesses of 135-kVp DECT images ranged from 2.0 to 4.0 mm (in 3 cases measurements were taken in a bone kernel image at 135-kVp because of a technical problem). MRI SI were measured in four ROIs placed as follows: anterior AF, centre of NP, posterior AF, and cerebrospinal fluid (CSF) (Supplementary Fig. 4). Sequences measured included STIR, TIRM, T2 or T2 TSE, depending on the available image data. Given the variability among the included MRI scanners, we applied an SI normalization technique to minimize the effects of scanner-related variations, enhancing the comparability and reliability of quantitative measurements derived from MRI SI data. Specifically, we normalized the SI of ROIs placed within IVDs against those of CSF (normalized ROI $= \frac{IVD ROI SI}{{\text{CSF ROI SI}}}$), which serves as a frequently used SI reference to balance magnetic field inhomogeneities^23–29^. The CSF ROI was placed within the anterior aspect of the dural sac, deliberately avoiding the cauda equina nerve roots to sample homogeneous fluid and to minimize partial-volume contamination. ROI placement in cMaps, 135-kVp DECT images and MRI was performed by a single observer at anatomically corresponding locations. In addition, ROI placement was documented visually alongside the recorded measurements. ROI positioning was checked in a randomly selected subset of the included patients by a second observer. A formal interobserver reliability analysis for ROI placement was not performed.

All measured HU and SI values were exported using the “Export ROI” plugin and compiled in an Excel file (Microsoft, Office365 Microsoft Corp., Redmond, WA, USA ,) for further analysis.

### Statistical analysis

Because of the clustered structure of the data, we chose a linear mixed model, which is commonly useful in analysing data from repeated measurements while appropriately handling its correlated structure^30,31^. A linear mixed model was used to describe the correlation of cMap values of AF ((anterior AF + posterior AF) /2) and NP (HU) with the corresponding PF-grade assigned to the IVD. The same analysis was performed for the 135 kVp DECT and MRI data, analysing the DECT HU/MRI SI value of the AF and the NP to the PF-grade. The linear mixed model analysis included PF-grade, IVD level and sex as fixed factors. The subject identifier was used as a random factor to determine intersubject variability. The covariance structure was estimated by variance components. Dependent variables in the separate analyses were: AF/NP cMap HU values, AF/NP DECT HU values, and IVD MRI signal intensities in AF and NP. PF-grade I and the IVD level L5-S1 were defined as the reference for all statistical comparisons. We excluded age as a fixed factor because of its strong correlation with PF-grade (Spearman r = 0.7), which introduced significant multicollinearity and thereby resulting in compromised reliability of estimates within the chosen model. Because of this substantial correlation, inclusion of both variables as independent factors in the selected model is not suitable according to established scientific guidelines^32^. MRI rating interrater reliability was calculated using weighted Cohen’s Kappa and comparing the results of the three readers for each IVD level included in a pairwise fashion (reader 1 with reader 2, reader 1 with reader 3, and reader 2 with reader 3). The arithmetic mean of all pairwise Cohen’s Kappa’s values was then calculated and used as the final interrater reliability. The final grading of all discs was then used for further evaluation. Agreement was interpreted to be excellent for κ > 0.80, good for κ = 0.60–0.79, moderate for κ = 0.40–0.59, fair for κ = 0.20–0.39, and slight for κ < 0.2. Statistical analysis was performed using SPSS version 29.0 (IBM Corp., Armonk, NY, USA). A *p*-value < 0.05 was considered statistically significant. Because of the exploratory nature of the study, no adjustment was made for multiplicity of *p*-values and confidence intervals. Therefore, all results are interpreted in an exploratory, hypothesis-generating manner.

## Results

### Patient population and Pfirrmann grading

In this study, we included 97 patients (53 women, 44 men; overall mean age 59.1 years, SD. 19.3) with 339 discs in total. DECT and MRI examinations were performed a median of 1 day apart (IQR 0–4.5 days; range 0–102 days); 44 of 97 patients (45.4%) underwent both examinations on the same day and 80 patients (82.5%) within 7 days. The longest interval was 102 days. PF-grades were distributed as follows: 18 grade I, 146 grade II, 102 grade III, 67 grade IV, and 6 grade V (Fig. 4). Disc levels were distributed as follows: (51) T12-L1, (54) L1–L2, (63) L2–L3, (61) L3–L4, (53) L4–L5, and (57) L5–S1. PF-grade II was most frequent at all IVD levels analysed (Supplementary Fig. 3). Mean interrater agreement for MRI Pfirrmann grading was good at κ = 0.6 (SD. 0.1, min. 0.3, max. 0.84).

### Disc density in cMap

Mean disc collagen and chondroitin density in cMaps separated by PF-grade was as follows: PF-grade I = 123.0 HU, SD. 27.1 (min. 83.7, max. 184.6); PF-grade II = 121.3 HU, SD. 27.1 (min. 25.7, max. 231.6); PF-grade III = 112.9 HU, SD. 30.3 (min. 37.5, max. 207.6); PF-grade IV = 107.3 HU, SD. 27.7 (min. 50.9, max. 161.2); and PF-grade V = 87.3 HU, SD. 29.7 (min. 45.5, max. 119.5). Mean disc density was highest at the L5-S1 level in both male and female patients: 134.2 HU (SD. 34.0) in men and 121.4 (SD. 40.9) in women (Supplementary Tables 4 and 5).

### Correlation of disc densities with PF-grades in cMap, 135-kVp DECT images, and MRI

**Fig. 2:**
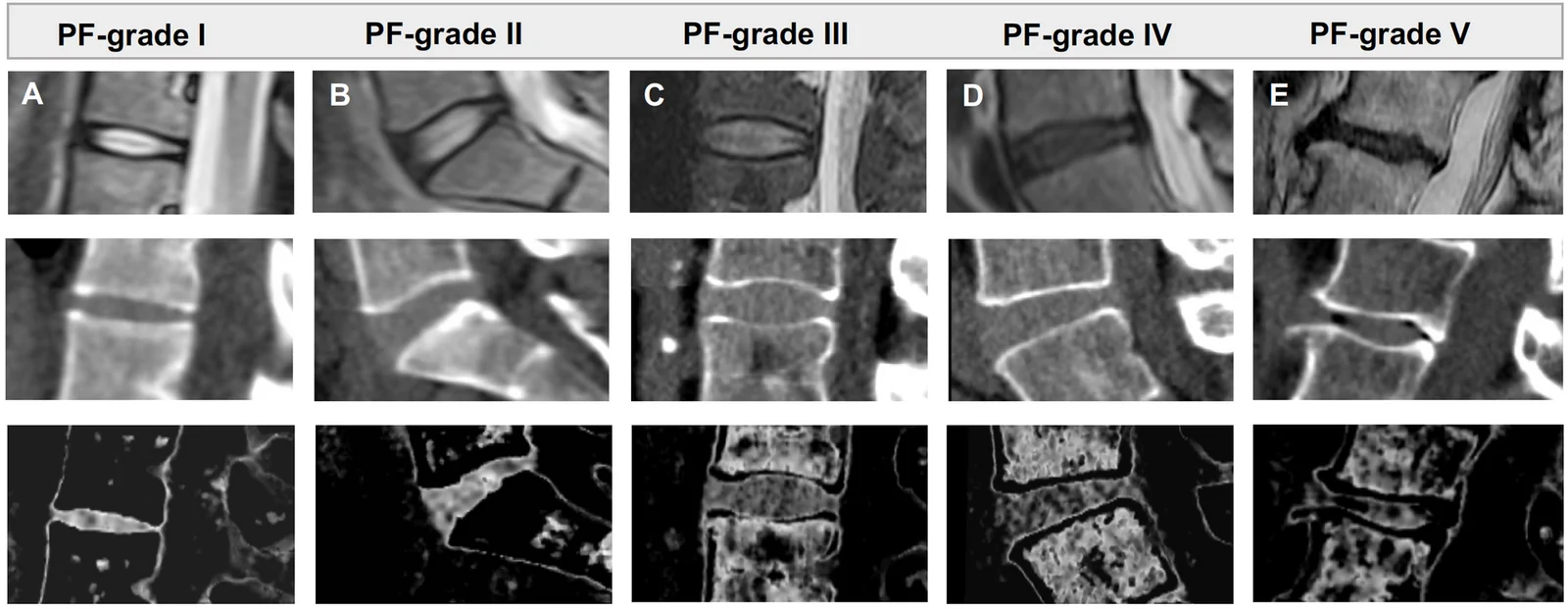
(**A**, **B**) Boxplot AF and NP density in cMap and DECT 135-kVp. Boxplot (**A**) shows AF and NP collagen and chondroitin density (HU) separated by PF-grade in DECT cMaps and in (**B**) at 135-kVp. Density of AF and NP decreased with increased PF-grade in DECT cMaps. Lowest disc density in AF and NP presets at PF-grade V. Compared to 135-kVp DECT images no density reduction is observed in the different disc compartments. Disc density in AF and NP scatters around 100 HU. AF, anulus fibrosus; NP, nucleus pulposus; DECT, dual-energy computed tomography

**Fig. 3:**
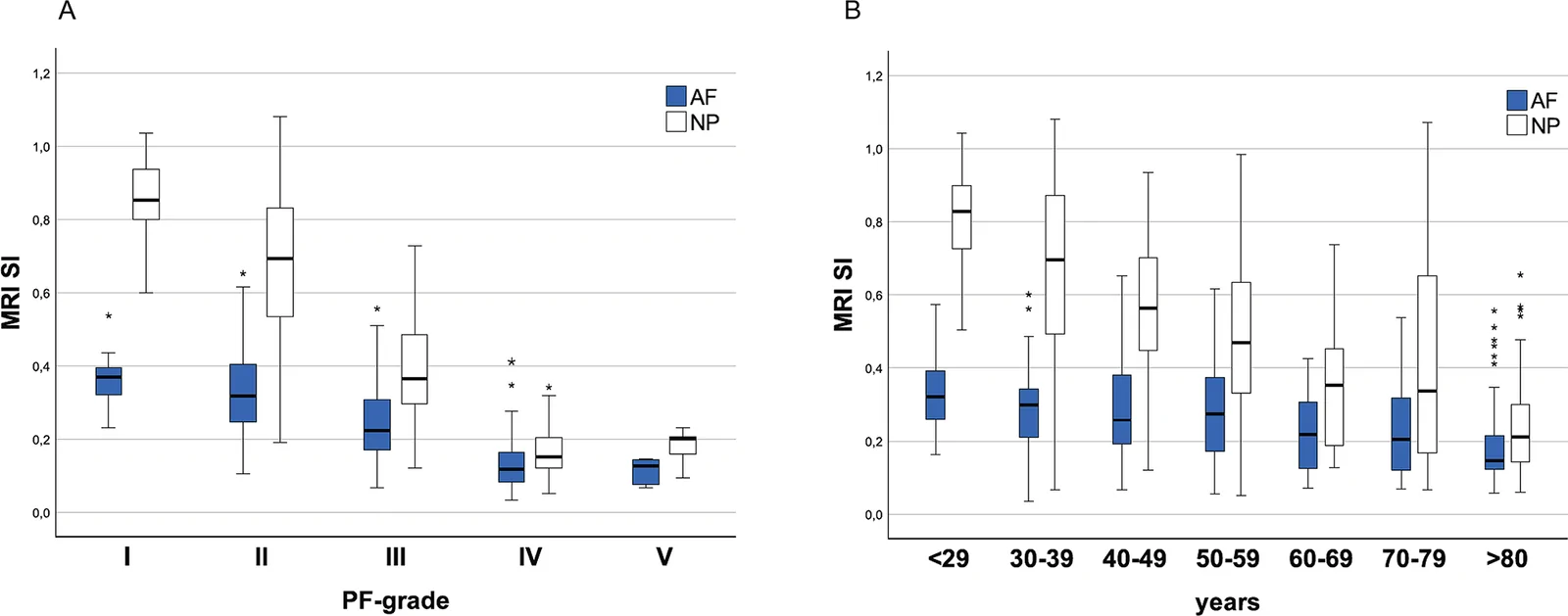
(**A**, **B**) Boxplot MRI AF and NP SI values. Boxplot (**A**) shows MRI SIs in AF and NP separated by PF-grades. Boxplot (**B**) shows MRI SIs in AF and NP separated by different age groups. SI decreases from PF-grade I to V, with a greater drop in SI values in the NP. With the age increasing, SI in both AF and NP reduces, NP values show a greater reduction in different age groups. AF, anulus fibrosus; NP, nucleus pulposus; SI, signal intensity; MRI, magnetic resonance imaging

In DECT cMaps, mixed model analysis revealed a difference in AF collagen and chondroitin density between discs graded as PF-grade V and discs graded as PF-grade I: AF in discs with PF-grade V had a lower collagen and chondroitin density compared to discs with PF-grade I (MD = − 31.1 HU (95% CI − 59.5 to − 2.7)), *p* = 0.032. Furthermore, the analysis showed a decrease in AF collagen and chondroitin density from PF Grade I to PF Grade IV (Table 1, Supplementary Fig. 2). Conversely, in 135-kVp DECT images, mixed model analysis found no difference in AF HU units and corresponding PF-grade, but a trend towards density reduction was apparent (Fig. 2). In MRI, a reduction was noticed in AF SI from PF-grade III to V compared to PF-grade I: MD in PF-grade III = − 0.1 (95% CI − 0.1 to 0.0), *p* = 0.003; MD in PF-grade IV = − 0.2 (95% CI − 0.2 to − 0.1), *p* =  < 0.001; MD in PF-grade V = − 0.2 (95% CI − 0.3 to − 0.1), *p* =  < 0.001 (Fig. 3). For NP densities in cMaps, the analysis revealed a density reduction from PF-grade I discs to PF-grade V discs (MD = − 37.8 (95% CI − 73.6 to − 2.0)), *p* = 0.038 (Fig. 2). A trend towards density reduction from PF-grade I to IV was observed, while the same density was found for PF-grades III and IV (111.40 HU) as compared with PF-grade I. For 135-kVp DECT, analysis revealed a difference in HU values between PF-grades I and IV (MD = 9.4 HU (95% CI 0.1 to 18.8), *p* = 0.048 (supplementary tables 1 and 3). Regarding NP MRI SI values, a decrease from PF-grade II to V compared to PF-grade I was visible: MD PF-grade II = − 0.1 (95% CI − 0.2 to 0.0), *p* = 0.013; MD PF-grade III = − 0.5 (95% CI − 0.5 to − 0.3), *p* =  < 0.001; PF-grade IV = − 0.5 (95% CI − 0.6 to − 0.4), *p* =  < 0.001; PF-grade V = − 0.5 (95% CI − 0.7 to − 0.4), *p* =  < 0.001.

**Table 1:** Linear mixed model assessing change in AF and NP in DECT cMap.

Parameter	Estimate	Sig	95% Confidence Interval	
			Lower bound	Upper bound
Estimates of fixed effects cMap AF				
Male	4.71	0.244	− 3.90	13.31
Female	Ref	Ref	Ref	Ref
PF-grade I	Ref	Ref	Ref	Ref
PF-grade II	0.35	0.968	− 17.11	17.81
PF-grade III	− 6.02	0.512	− 24.10	12.06
PF-grade IV	− 7.17	0.454	− 26.03	11.68
PF-grade V	− 31.11	0.032	− 59.52	− 2.71
Estimates of fixed effects cMap NP				
Male	5.43	0.367	− 6.48	17.34
Female	Ref	Ref	Ref	Ref
PF-grade I	Ref	Ref	Ref	Ref
PF-grade II	− 6.36	0.575	− 28.67	15.95
PF-grade III	− 1.98	0.867	− 25.24	21.28
PF-grade IV	− 1.98	0.873	− 26.35	22.38
PF-grade V	− 37.81	0.038	− 73.59	− 2.04

Comparing males and females, a difference in AF density in male by 4.71 HU and NP density by 5.43 HU is visible. A statistically significant difference between PF-grade I and PF-grade V in AF (MD = − 31.11 HU, *p* = 0.032 ) as well as in NP (MD = − 37.81 HU, *p* = 0.038) is visible. The analysed data included PF-grade distribution as follows: PF-grade I (*n* = 18), PF-grade II (*n* = 146), PF-grade III (*n* = 102), PF-grade IV (*n* = 67), PF-grade V (*n* = 6).

AF, anulus fibrosus; NP, nucleus pulposus; DECT, dual-energy computed tomography; Ref., reference level for comparison.

Regarding sex differences, cMaps showed numerically higher AF and NP density in men (by 4.7 and 5.4 HU, respectively) compared with women (Table 1, 2,Supplementary Fig. 1). In 135-kVp DECT scans, AF and NP had nearly the same density in men and women, with AF density being 0.2 HU higher and 0.8 HU lower in NP in men. MRI SIs values in AF and NP were higher in male patients (AF: MD = 0.04, *p* = 0.025; NP: MD = 0.1, *p* = 0.008) (Supplementary Tables 2 and 3).

**Table 2:** Linear mixed model estimating mean AF and NP density in DECT cMap separated by PF-grade.

PF-grade	Mean	95% confidence interval	
		Lower bound	Upper bound
Estimates PF-grade cMap AF			
I	120.74	103.63	137.84
II	121.09	115.01	127.17
III	114.72	108.48	120.96
IV	113.56	105.50	121.62
V	89.62	66.95	112.29
Male	114.30	106.30	122.30
Female	109.59	102.00	117.18
Estimates PF-grade cMap NP			
I	113.64	91.48	135.80
II	107.02	98.88	115.16
III	111.40	103.27	119.52
IV	111.39	100.92	121.87
V	75.57	47.47	103.66
Male	106.47	95.78	117.15
Female	101.04	90.96	111.11

A density reduction in AF and NP HU values from PF-grade I to V is shown. A difference in mean density between male and female patients is visible.

AF, anulus fibrosus; NP, nucleus pulposus; DECT, dual-energy computed tomography.

## Discussion

### Summary

**Fig. 4:**
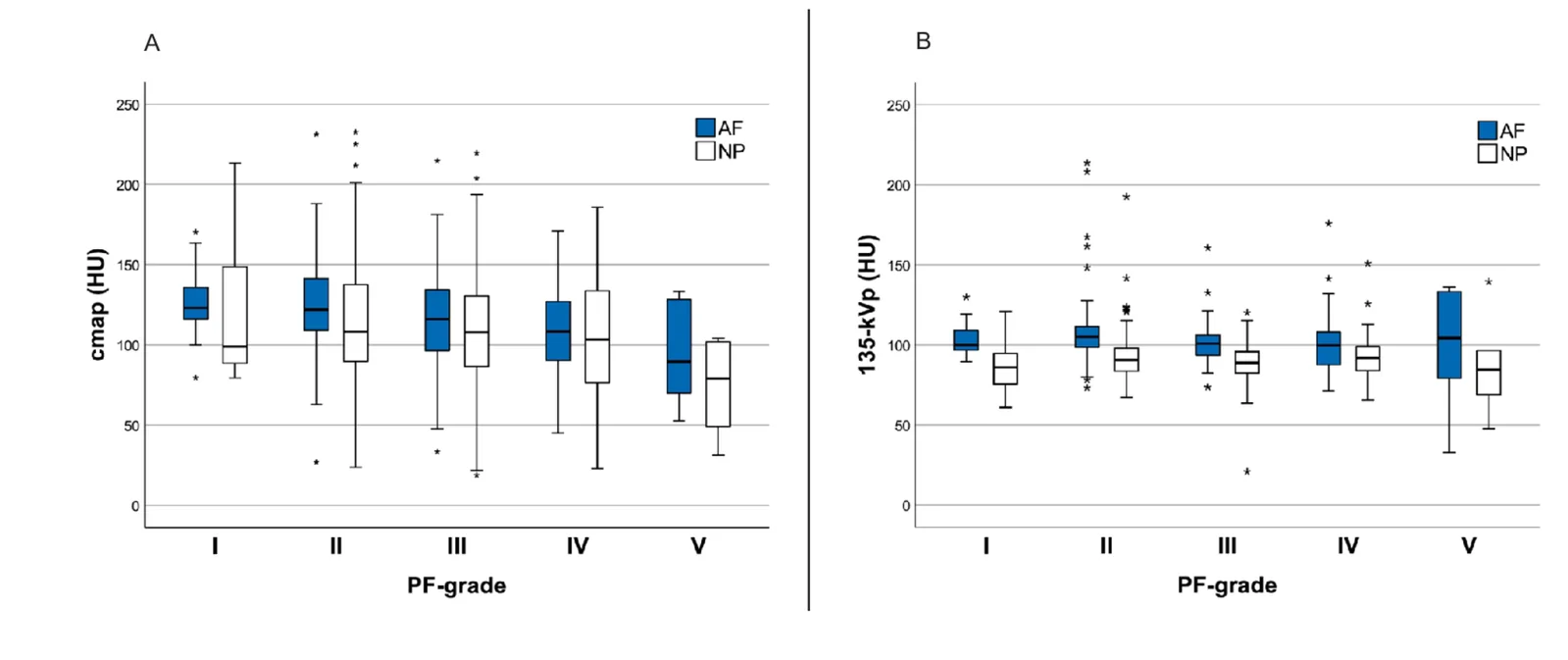
Morphology of intervertebral discs in different imaging qualities separated by PF-grades. Images correspond to five different patients. Figure showing different PF-grade I to V in MRI, DECT and cMaps. Column A (female; T12-L1): MRI (STIR), 135-kVp DECT: 113.44 HU and cMap: 146.51 HU. B (female; L5-S1): MRI (STIR), 135-kVp DECT: 87.01 HU and cMap: 115.27 HU. C (female; L3-L4): MRI (T2_TIRM), 135-kVp DECT: 92.13 HU and cMap: 111.62 HU. D (male; L3-L4): MRI (T2_TSE), 135-kVp DECT: 86.34 HU and cMap: 109.92 HU. E (female; L1-L2): MRI (T2) 135-kVp DECT :75.86 HU and cMap: 103.19 HU. MRI, magnetic resonance imaging; DECT, dual-energy computed tomography

To our knowledge, this is the first study to quantitatively evaluate whether dual-energy computed tomography (DECT) collagen maps (cMaps) can determine the severity of intervertebral disc degeneration (IDD) using MRI as the reference standard. Our results demonstrate a reduction in mean intervertebral disc (IVD) collagen and chondroitin density with increasing Pfirrmann (PF) grade. This degenerative density loss was significant in both the annulus fibrosus (AF) and nucleus pulposus (NP) when comparing PF-grade I to PF-grade V discs and was more pronounced in cMaps than in 135-kVp DECT images. Additionally, sex-related differences were observed, with men showing numerically higher density values in both the AF and NP. These findings may reflect not only general fluid loss, as indicated by T2-weighted MRI, but also a broader disruption of disc microstructure, likely driven by the depletion of proteoglycans and collagen. This interpretation aligns with previous research using advanced quantitative MRI techniques, such as glycosaminoglycan chemical exchange saturation transfer (gagCEST) imaging, and can now, for the first time, be directly correlated with DECT measurements. These observations suggest that DECT cMap imaging could provide valuable soft tissue information and serve as an additional tool for IVD classification. It offers a cost-effective method for evaluating disc microstructure, potentially applied opportunistically during other imaging studies (e.g., abdominal CT scans) or for patients without access to MRI diagnostics. With the recent integration of photon-counting detector technology into clinical practice, wider availability of this diagnostic approach is anticipated (Fig. 4).

### Comparison of published results

Over the last decade, DECT imaging has gained acceptance in various clinical settings because it allows for specific material decomposition based on the effective atomic number of different tissues. Collagen maps, specifically, provide spatially resolved information about collagen distribution by quantifying the heavy side chains of hydroxyproline and hydroxylysine, which are key components for the strength and stability of collagen-rich tissues^11,33,34^. The study by Bai et al. confirmed the visualization of hydroxyproline and hydroxylysine changes in colour-coded DECT- collagen mapping^35^. A recent study revealed the contribution of chondroitin sulphate and collagen to the density’s measurements in DECT imaging^12^. Previous proof-of-concept work by Pohlan et al. demonstrated the technical feasibility of DECT collagen mapping using ex vivo specimens with artificially created lesions, establishing the foundation for clinical translation of this technology^36^. To our knowledge there is a lack of literature correlating histological findings with DECT cMap collagen and chondroitin measurements, which should be investigated in future studies.

In the field of musculoskeletal imaging, while a few studies have used DECT cMaps to analyse both IVD morphology and degenerative changes^12,36–39^, only a limited number have compared these findings with PF-grades^17,18^. Recent studies by Mahmoudi et al. and Bernatz et al. focused on the qualitative, morphological assessment of IVDs using colour-coded cMaps, reporting high diagnostic accuracy and excellent interrater reliability^17,18^. Mahmoudi et al. found an overall accuracy of DECT cMaps of 85.5% with excellent interrater agreement of 0.9 for evaluation of lumbar disc degeneration based on Pfirrmann grading^18^. Similarly, Bernatz et al. evaluated 612 thoracic IVDs in 51 patients, reporting an overall diagnostic accuracy of 81% with excellent interrater reliability (ICC = 0.89)^17^. Our study complements this work by providing objective quantitative measurements that eliminate inter-observer variability inherent in visual assessment method.

Our sequential dual-energy acquisition differs from the third-generation dual-source systems with tin filtration used by Mahmoudi et al. and Bernatz et al.^17,18^. Sequential scanning is one of the three established dual-energy strategies alongside dual-source and rapid kV-switching, which differ mainly in spectral separation^40^. Spectral separation is not the sole determinant of quantitative accuracy: a sequential implementation ranked within the statistically most accurate group for iodine quantification among seven systems in an intermanufacturer phantom study^41^. This phantom performance may not directly translate to clinical practice, where patient motion or differences in contrast timing between the two acquisitions may degrade material decomposition. In our setting, however, the thoracolumbar spine represents a static, non-enhancing target, so that this limitation is largely avoided. While these data refer to iodine rather than collagen decomposition, collagen-specific decomposition has already been applied successfully in musculoskeletal imaging^42^ and, using the same acquisition technique, in the lumbar spine^21,22^. Quantitative values nevertheless remain platform-specific and require external validation, as likewise acknowledged by the comparison studies^17,18^. This spectral dependence also applies to the 135-kVp DECT image reference used here, rather than the 120 kVp commonly employed in routine lumbar CT. Because soft tissue is radiologically water-equivalent and shows no relevant tube-voltage dependence between 70 and 140 kV, unlike bone and adipose tissue^43^, and because our analysis compared Pfirrmann grades rather than absolute attenuation values, the small difference from a 120-kVp protocol should not affect our findings.

Assessing degenerative changes as early as possible would be very beneficial in case of disc degeneration. In our study, however, cMap analysis did not show significant differences between PF-grades II, III, and IV. This could be explained by the fact that PF-grades derive primarily from T2 signal intensity, which mainly reflect water content, meaning that cMap and the PF-grades do not assess entirely the same tissue property, together with the small subgroup sizes or interindividual value scattering or other IVD components that may have impacted the presented measurements. Moreover, a water-content-based reference may be suboptimal for validating collagen- and chondroitin-sensitive DECT measurements. Future studies could address this using larger, balanced subgroups and a compositionally specific reference such as gagCEST, T1ρ or T2 mapping, acknowledging that long acquisition times and limited availability may currently restrict their routine use. This limited availability applies to dual-energy CT as well, although CT infrastructure across Europe has expanded, with scanner numbers increasing in most EU countries^44^, and photon-counting detector CT, now entering clinical practice, resolves the energy of every detected photon and therefore provides spectral data at every acquisition, inherently enabling material decomposition^45–47^.

Further studies have demonstrated that DECT imaging yields more diagnostic information in the detection of disc injury^37^ and provides better interrater agreement for the detection of disc displacement^22^ compared to 135-kVp DECT images. Moreover, Abdellatif et al. demonstrated superior specificity and accuracy in the diagnosis of lumbar disc extrusion and sequestration for 135-kVp DECT images supplemented by DECT cMaps compared with 135-kVp DECT images alone^39^. Other DECT applications have also shown promise. Further supporting the clinical utility of DECT with another form of mapping, Basavaraju et al. for instance, demonstrated that Electron-density (ED) mapping significantly outperforms standard CT in detecting lumbar disc herniations and nerve-root impingement^6^. Compared to cMaps, ED-mapping is based on the determination of the effective atomic number of the analysed material. While both approaches utilize dual-energy technology, the three-material decomposition method applied here specifically targets collagen quantification, addressing different aspects of disc composition assessment. In our cohort, we observed that disc density in cMaps was higher in male patients. This may be attributed to the higher mean age of women in our study population (63.5 vs. 53.8 years), aligning with previous findings of age-related density loss^20^, although those results were not sex- specific. We also found that the mean cMap IVD density was highest at the L5-S1 level in both men and women. This finding is consistent with the results in prior research^12^ and may be explained by the higher level of pressure that acts on this disc level compared to others.

In 135-kVp DECT images, a significant difference between NP HU values in PF-grades I to IV was noticed, which might be explained by the fact that NP values with PF-grade IV (93.5 HU, SD 14.4) presented the highest mean HU measurements compared to all other PF-grades. Our analysis of MRI signal intensity (SI) data showed a significant reduction in AF and NP values in more advanced degeneration (PF-grades III to V), which aligns with previous reports that T2 SI values decrease with IDD^26,27,48^. This SI reduction was slightly more prominent in NP than in AF, reflecting the more pronounced water- and proteoglycan loss that predominantly occurs in the NP^49,50^.

Regarding the MRI Pfirrmann grading, the mean interrater agreement in our study was good at κ = 0.6, which is consistent with reliability scores reported in the literature, ranging from κ = 0.49 to 0.83^51–53^. In the field of quantitative MRI imaging, more recent methods, such as T2 mapping, diffusion-weighted MRI with apparent diffusion coefficient mapping, T1ρ mapping, or gagCEST can provide additional information regarding biochemical changes occurring in early degeneration^54–57^. Compositionally specific techniques such as gagCEST are clearly valuable and would be well suited to the planning of dedicated future studies rather than to a pragmatic, routine-based design such as ours.

### Limitations

Several important limitations should be considered when interpreting our findings. Our retrospective approach allowed us to analyse a large number of cases, but it also introduced potential selection bias due to the use of preexisting patient data with the possibility of an inhomogeneous patient collective. Our patient population was heterogeneous, showing an uneven distribution of degeneration grades, with very few severely degenerated (PF-grade V) discs. Additionally, our findings from thoracolumbar spine discs cannot be assumed to apply equally to the upper and mid-thoracic spine discs. However, the substantial sample size in our study enabled robust statistical analysis, and all image assessments were performed by experienced radiologists with good interrater reliability, supporting the validity of our results.

To address technical and imaging limitations the following must be considered.

Dual-energy CT scanners are not yet available in all centres, regions and countries, which may limit the generalizability and the clinical implementation of the approach described here. In addition, absolute attenuation values obtained at 135-kVp DECT images are not directly interchangeable with those from a standard 120-kVp protocol.

The MRI images in our study came from different scanners using various sequences, which introduced some variability in image quality. This heterogeneity might have affected both signal intensity measurements and MRI rating performance. However, we addressed this by normalizing signal intensities to minimize the effects of scanner-related variation. Importantly, this variability reflects typical clinical practice. Several limitations relate to the placement of the regions of interest (ROIs), which required careful adaptation to the individual shape and degenerative state of each disc. In severely degenerated discs, cases in which reliable measurements were not possible due to limited disc space or potential interference from surrounding bone structures were excluded in order to avoid partial-volume effects. Nevertheless, a residual influence of partial-volume effects from adjacent bone structures cannot be excluded, even with standardized measurement procedures. Although the formal loss of distinction between the annulus fibrosus (AF) and nucleus pulposus (NP) in advanced degeneration, we positioned our measurements based on expected anatomical locations. Our results confirmed that these structural compartments are indeed no longer clearly maintained structurally on imaging in advanced disease. Regarding the CSF ROI, although it was positioned within the anterior aspect of the dural sac to minimize partial-volume contamination, a minimal influence to the measured CSF signal cannot be fully excluded. Finally, all ROIs were placed by a single observer. Although ROI positioning was reviewed in a randomly selected subset of the included patients to confirm consistent and anatomically correct placement, no formal interobserver reliability analysis was performed.

Our results should be validated through prospective studies with larger, more balanced patient groups that can better examine how factors such as age, sex, and body mass index influence IDD.

### Conclusion

These data indicate that quantitative analysis of cMaps reflects collagen and chondroitin loss in relation to PF-grades in disc degeneration, especially showing a prominent loss of collagen and chondroitin density with increasing PF-grades. DECT cMaps could be an additional tool for IVD classification. Quantitative DECT imaging of IVD should be investigated further in larger cohorts.

## Supplementary Information

Below is the link to the electronic supplementary material.

Supplementary Material 1

Supplementary Material 2

Supplementary Material 3

Supplementary Material 4

Supplementary Material 5

Supplementary Material 6

Supplementary Material 7

Supplementary Material 8

Supplementary Material 9

Supplementary Material 10

Supplementary Material 11

## Data Availability

Data will be made available upon reasonable request to the corresponding author. Due to strict German regulations along with the patient data policy it is not possible to make all data publicly available.

## Abbreviations

AF: Annulus fibrosus
CI: Confidence Interval
cMap: Collagen map
CSF: Cerebrospinal fluid
DECT: Dual-energy computed tomography
gagCEST: Chemical exchange saturation transfer imaging of glycosaminoglycan content
HU: Hounsfield unit
IDD: Intervertebral disc degeneration
IVD: Intervertebral disc
MD: Mean difference
NP: Nucleus pulposus
PF-grade: Pfirrmann grade
ROI: Region of interest
SI: Signal intensity
STIR: Short-tau inversion recovery
T: Tesla
TSE: Turbo-spin echo
TIRM: turbo inversion recovery magnitude

## References

[1] Pfirrmann, C. W., Metzdorf, A., Zanetti, M., Hodler, J. & Boos, N. Magnetic resonance classification of lumbar intervertebral disc degeneration. *Spine (Phila Pa 1976)* **26**(17), 1873–1878 (1976). 10.1097/00007632-200109010-00011 11568697

[2] Russo, R. J. et al. Assessing the risks associated with MRI in patients with a pacemaker or defibrillator. *N. Engl. J. Med.* **376**(8), 755–764 (2017). 28225684 10.1056/NEJMoa1603265

[3] Dalrymple, N. C., Prasad, S. R., Freckleton, M. W. & Chintapalli, K. N. Informatics in radiology (infoRAD): Introduction to the language of three-dimensional imaging with multidetector CT. *Radiographics* **25**(5), 1409–1428 (2005). 16160120 10.1148/rg.255055044

[4] Koch, V. et al. Assessment of thoracic disk herniation by using virtual noncalcium dual-energy CT in comparison with standard grayscale CT. *Eur. Radiol.* **31**(12), 9221–9231 (2021). 34076743 10.1007/s00330-021-07989-5 PMC8589804

[5] Shinohara, Y. et al. Evaluation of lumbar intervertebral disc degeneration using dual energy CT virtual non-calcium imaging. *Eur. J. Radiol.* **124**, 108817 (2020). 31931302 10.1016/j.ejrad.2020.108817

[6] Basavaraju, U. K., Balol, S., Manohar, V. & Naik, Y. Diagnostic performance of dual-energy CT with electron-density reconstruction for lumbar disc herniation. *SA J. Radiol.* **28**(1), 3000 (2024). 39650705 10.4102/sajr.v28i1.3000 PMC11622118

[7] Le Maitre, C. L., Pockert, A., Buttle, D. J., Freemont, A. J. & Hoyland, J. A. Matrix synthesis and degradation in human intervertebral disc degeneration. *Biochem. Soc. Trans.* **35**(4), 652–655 (2007). 17635113 10.1042/BST0350652

[8] Liang, H. et al. The proteolysis of ECM in intervertebral disc degeneration. *Int. J. Mol. Sci.* **23**(3), 1715 (2022). 35163637 10.3390/ijms23031715 PMC8835917

[9] Lyons, G., Eisenstein, S. M. & Sweet, M. B. Biochemical changes in intervertebral disc degeneration. *Biochim. Biophys. Acta.* **673**(4), 443–453 (1981). 7225426 10.1016/0304-4165(81)90476-1

[10] Roughley, P. J. Biology of intervertebral disc aging and degeneration: Involvement of the extracellular matrix. *Spine (Phila Pa 1976)* **29**(23), 2691–2699 (2004). 15564918 10.1097/01.brs.0000146101.53784.b1

[11] Johnson, T. R. C. et al. Material differentiation by dual energy CT: Initial experience. *Eur. Radiol.* **17**(6), 1510–1517 (2007). 17151859 10.1007/s00330-006-0517-6

[12] Pohlan, J. et al. Age-dependent microstructural changes of the intervertebral disc: A validation of proteoglycan-sensitive spectral CT. *Eur. Radiol.* **31**(12), 9390–9398 (2021). 33993329 10.1007/s00330-021-08028-z PMC8589800

[13] Hu, B. et al. Inflammatory microRNA-194 and -515 attenuate the biosynthesis of chondroitin sulfate during human intervertebral disc degeneration. *Oncotarget* **8**(30), 49303–49317 (2017). 28514734 10.18632/oncotarget.17571 PMC5564769

[14] Deng, K., Sun, C., Liu, C. & Ma, R. Initial experience with visualizing hand and foot tendons by dual-energy computed tomography. *Clin. Imaging* **33**(5), 384–389 (2009). 19712820 10.1016/j.clinimag.2008.12.007

[15] Fickert, S. et al. Assessment of the diagnostic value of dual-energy CT and MRI in the detection of iatrogenically induced injuries of anterior cruciate ligament in a porcine model. *Skeletal. Radiol.* **42**(3), 411–417 (2013). 22923156 10.1007/s00256-012-1500-8

[16] Sun, C. et al. An initial qualitative study of dual-energy CT in the knee ligaments. *Surg. Radiol. Anat.* **30**(5), 443–447 (2008). 18427711 10.1007/s00276-008-0349-y

[17] Bernatz, S. et al. Assessment of thoracic disc degeneration using dual-energy CT-based collagen maps. *Eur. Radiol. Exp.* **8**(1), 95 (2024). 39186171 10.1186/s41747-024-00500-x PMC11347509

[18] Mahmoudi, S. et al. Potential of dual-energy CT-based collagen maps for the assessment of disk degeneration in the lumbar spine. *Acad. Radiol.* **31**(9), 3732–3739 (2024). 38519304 10.1016/j.acra.2024.02.036

[19] Deppe, D. et al. Dual-energy-CT for osteitis and fat lesions in axial spondyloarthritis: How feasible is low-dose scanning?. *Diagnostics* **13**(4), 776 (2023). 36832264 10.3390/diagnostics13040776 PMC9955853

[20] Diekhoff, T. et al. Single-source dual-energy computed tomography for the assessment of bone marrow oedema in vertebral compression fractures: A prospective diagnostic accuracy study. *Eur. Radiol.* **29**(1), 31–39 (2019). 29948088 10.1007/s00330-018-5568-y

[21] Stelbrink, C. et al. Diagnostic accuracy of dual energy computed tomography for suspected pyogenic spondylodiscitis. *Sci. Rep.* **15**(1), 20040 (2025). 40494906 10.1038/s41598-025-04216-9 PMC12152176

[22] Schömig, F. et al. Vertebral disk morphology of the lumbar spine: A retrospective analysis of collagen-sensitive mapping using dual-energy computed tomography. *Skeletal. Radiol.* **50**(7), 1359–1367 (2021). 33277674 10.1007/s00256-020-03685-5 PMC8119261

[23] Wahid, K. A. et al. Intensity standardization methods in magnetic resonance imaging of head and neck cancer. *Phys. Imaging Radiat. Oncol.* **20**, 88–93 (2021). 34849414 10.1016/j.phro.2021.11.001 PMC8607477

[24] Stamoulou, E. et al. Harmonization strategies in multicenter MRI-based radiomics. *J. Imaging*10.3390/jimaging8110303 (2022). 36354876 PMC9695920

[25] Luoma, E. K. et al. Suitability of cerebrospinal fluid as a signal-intensity reference on MRI: Evaluation of signal-intensity variations in the lumbosacral dural sac. *Neuroradiology* **39**(10), 728–732 (1997). 9351111 10.1007/s002340050496

[26] Nagashima, M. et al. A method for quantifying intervertebral disc signal intensity on T2-weighted imaging. *Acta Radiol.* **53**(9), 1059–1065 (2012). 22993271 10.1258/ar.2012.120039

[27] Murto, N., Luoma, K., Lund, T. & Kerttula, L. Reliability of T2-weighted signal intensity-based quantitative measurements and visual grading of lumbar disc degeneration on MRI. *Acta Radiol.* **64**(6), 2145–2151 (2023). 37078166 10.1177/02841851231169079

[28] Michopoulou, S., Costaridou, L., Vlychou, M., Speller, R. & Todd-Pokropek, A. Texture-based quantification of lumbar intervertebral disc degeneration from conventional T2-weighted MRI. *Acta Radiol.* **52**(1), 91–98 (2011). 21498333 10.1258/ar.2010.100166

[29] Fan, S.-W. et al. Quantitative MRI analysis of the surface area, signal intensity and MRI index of the central bright area for the evaluation of early adjacent disc degeneration after lumbar fusion. *Eur. Spine J.* **21**(9), 1709–1715 (2012). 22526697 10.1007/s00586-012-2293-0 PMC3459106

[30] Schober, P. & Vetter, T. R. Linear mixed-effects models in medical research. *Anesth. Analg.* **132**(6), 1592–1593 (2021). 34032662 10.1213/ANE.0000000000005541

[31] Schober, P. & Vetter, T. R. Repeated measures designs and analysis of longitudinal data: If at first you do not succeed-try, try again. *Anesth. Analg.* **127**(2), 569–575 (2018). 29905618 10.1213/ANE.0000000000003511 PMC6072386

[32] Shieh, Y.-Y. & Fouladi, R. T. The effect of multicollinearity on multilevel modeling parameter estimates and standard errors. *Educ. Psychol. Meas.* **63**(6), 951–985 (2003).

[33] Guenther, H. L., Guenther, H. E. & Fleisch, H. The influence of 1-hydroxyethane-1,1-diphosphonate and dichloromethanediphosphonate on lysine hydroxylation and cross-link formation in rat bone, cartilage and skin collagen. *Biochem. J.* **196**(1), 303–310 (1981). 6458288 10.1042/bj1960303 PMC1162994

[34] Murad, S. et al. Regulation of collagen synthesis by ascorbic acid. *Proc. Natl. Acad. Sci. U. S. A.* **78**(5), 2879–2882 (1981). 6265920 10.1073/pnas.78.5.2879 PMC319462

[35] Bai, R., He, X. & Huang, J. A basic study for the molecular imaging of dual-energy CT in diagnosing anterior cruciate ligament injury of knee joint. *Acta Radiol.* **64**(4), 1589–1599 (2022). 36357954 10.1177/02841851221135853

[36] Pohlan, J. et al. Visualizing patterns of intervertebral disc damage with dual-energy computed tomography: Assessment of diagnostic accuracy in an ex vivo spine biophantom. *Acta Radiol.* **63**(8), 1118–1125 (2022). 34219471 10.1177/02841851211025863 PMC9272519

[37] Pumberger, M. et al. Disk injury in patients with vertebral fractures-a prospective diagnostic accuracy study using dual-energy computed tomography. *Eur. Radiol.* **29**(8), 4495–4502 (2019). 30649597 10.1007/s00330-018-5963-4 PMC6610270

[38] Schomig, F. et al. Vertebral disk morphology of the lumbar spine: A retrospective analysis of collagen-sensitive mapping using dual-energy computed tomography. *Skeletal. Radiol.* **50**(7), 1359–1367 (2021). 33277674 10.1007/s00256-020-03685-5 PMC8119261

[39] Abdellatif, W. et al. Dual energy computed tomography collagen material decomposition for detection of lumbar spine disc extrusion and sequestration: A comparative study with greyscale computed tomography. *Can. Assoc. Radiol. J.* **74**(1), 110–118 (2023). 35948996 10.1177/08465371221118886

[40] Johnson, T. R. Dual-energy CT: General principles. *AJR Am. J. Roentgenol.* **199**(5 Suppl), S3-8 (2012). 23097165 10.2214/AJR.12.9116

[41] Jacobsen, M. C. et al. Intermanufacturer comparison of dual-energy CT iodine quantification and monochromatic attenuation: A phantom study. *Radiology* **287**(1), 224–234 (2018). 29185902 10.1148/radiol.2017170896

[42] Jeon, J. Y., Lee, S.-W., Jeong, Y. M. & Yu, S. The utility of dual-energy CT collagen material decomposition technique for the visualization of tendon grafts after knee ligament reconstruction. *Eur. J. Radiol.* **116**, 225–230 (2019). 31054789 10.1016/j.ejrad.2019.03.012

[43] Ma, X., Figl, M., Unger, E., Buschmann, M. & Homolka, P. X-ray attenuation of bone, soft and adipose tissue in CT from 70 to 140 kV and comparison with 3D printable additive manufacturing materials. *Sci. Rep.* **12**(1), 14580 (2022). 36028638 10.1038/s41598-022-18741-4 PMC9418162

[44] Eurostat. Healthcare resource statistics - technical resources and medical technology Luxembourg: Eurostat; 2026 [updated July 2026. Available from: https://ec.europa.eu/eurostat/statistics-explained/index.php?title=Healthcare_resource_statistics_-_technical_resources_and_medical_technology#Explore_further.

[45] Sartoretti, T., Wildberger, J. E., Flohr, T. & Alkadhi, H. Photon-counting detector CT: Early clinical experience review. *Br. J. Radiol.* **96**(1147), 20220544 (2023). 36744809 10.1259/bjr.20220544 PMC10321251

[46] Schwartz, F. R. et al. Photon-counting CT: Technology, current and potential future clinical applications, and overview of approved systems and those in various stages of research and development. *Radiology* **314**(3), e240662 (2025). 40067107 10.1148/radiol.240662 PMC11950899

[47] van der Bie, J. et al. Photon-counting CT: Review of initial clinical results. *Eur. J. Radiol.* 10.1016/j.ejrad.2023.110829 (2023). 37080060

[48] Salamat, S., Hutchings, J., Kwong, C., Magnussen, J. & Hancock, M. J. The relationship between quantitative measures of disc height and disc signal intensity with Pfirrmann score of disc degeneration. *Springerplus* **5**(1), 829 (2016). 27386278 10.1186/s40064-016-2542-5 PMC4917505

[49] Li, H., Yan, J.-z, Chen, Y.-j, Kang, W.-b & Huang, J.-x. Non-invasive quantification of age-related changes in the vertebral endplate in rats using in vivo DCE-MRI. *J. Orthop. Surg. Res.* **12**(1), 169 (2017). 29121960 10.1186/s13018-017-0669-x PMC5680764

[50] Marinelli, N. L., Haughton, V. M., Muñoz, A. & Anderson, P. A. T2 relaxation times of intervertebral disc tissue correlated with water content and proteoglycan content. *Spine* **34**(5), 520–524 (2009). 19247172 10.1097/BRS.0b013e318195dd44

[51] Arana, E. et al. Lumbar spine: Agreement in the interpretation of 1.5-T MR images by using the Nordic Modic Consensus Group classification form. *Radiology* **254**(3), 809–817 (2010). 20123897 10.1148/radiol.09090706

[52] Carrino, J. A. et al. Lumbar spine: Reliability of MR imaging findings. *Radiology* **250**(1), 161–170 (2009). 18955509 10.1148/radiol.2493071999 PMC2657480

[53] Urrutia, J. et al. The Pfirrmann classification of lumbar intervertebral disc degeneration: An independent inter- and intra-observer agreement assessment. *Eur. Spine J.* **25**(9), 2728–2733 (2016). 26879918 10.1007/s00586-016-4438-z

[54] Wu, N. et al. Comparison of apparent diffusion coefficient and T2 relaxation time variation patterns in assessment of age and disc level related intervertebral disc changes. *PLoS ONE* **8**(7), e69052 (2013). 23922680 10.1371/journal.pone.0069052 PMC3724871

[55] Watanabe, A. et al. Classification of intervertebral disk degeneration with axial T2 mapping. *AJR Am. J. Roentgenol.* **189**(4), 936–942 (2007). 17885068 10.2214/AJR.07.2142

[56] Takayama, Y. et al. T1ρ is superior to T2 mapping for the evaluation of articular cartilage denaturalization with osteoarthritis: Radiological-pathological correlation after total knee arthroplasty. *Eur. J. Radiol.* **82**(4), e192 (2013). 23265927 10.1016/j.ejrad.2012.11.031

[57] Xiong, X. et al. Multi-parameter evaluation of lumbar intervertebral disc degeneration using quantitative magnetic resonance imaging techniques. *Am. J. Transl. Res.* **10**(2), 444–454 (2018). 29511438 PMC5835809

