# Supplementary material for: Quantification of intervertebral disc degeneration using dual-energy computed tomography collagen maps: a comparative study with MRI Pfirrmann grading
Source: Sci Rep. 2026 Sep 3;16:27687. doi: 10.1038/s41598-026-69070-9 (PMC13542269; doi:10.1038/s41598-026-69070-9)
Supplement: Supplementary file 1 [file 41598_2026_69070_MOESM10_ESM.docx]

**Patient overlap with earlier departmental studies**

The 97 subjects analysed in the present study were drawn from four earlier studies conducted at our department, in which the same routinely acquired Dual-energy CT (DECT) were used to address different research questions. Because the recruitment periods of these studies overlap in part with the present study period (March 2015 – July 2021), individual patients are shared between cohorts.

Regarding the differences in scientific investigation, the mentioned studies examined the spine and intervertebral discs of patients using DECT-based virtual non-calcium images (studies 1 and 2). The first study focused on pathologies of the sacroiliac joints in patients with axial spondylarthritis, while the second study analyzed the diagnostic accuracy of dual-energy imaging of bone marrow edema in vertebral compression fractures. The third study concentrated on the disc morphology and detection of pathologies of the lumbar spine using DECT-based collagen mapping. The fourth study analyzes inflammatory disc disease and degenerative disc disease using DECT.

None of the earlier studies quantified collagen and chondroitin density within the intervertebral discs and correlated it with the MRI Pfirrmann grade.

**Source studies and number of shared subjects**

**1.** Deppe D, Ziegeler K, Hermann KGA, et al. Dual-Energy-CT for osteitis and fat lesions in axial spondyloarthritis: how feasible is low-dose scanning? Diagnostics 2023;13:776. <https://doi.org/10.3390/diagnostics13040776>

**Shared subjects: n = 36**

**2.** Diekhoff T, Engelhard N, Fuchs M, et al. Single-source dual-energy computed tomography for the assessment of bone marrow oedema in vertebral compression fractures: a prospective diagnostic accuracy study. Eur Radiol 2019;29:31–39. <https://doi.org/10.1007/s00330-018-5568-y>

**Shared subjects: n = 41**

**3.** Schömig F, Pumberger M, Palmowski Y, et al. Vertebral disk morphology of the lumbar spine: a retrospective analysis of collagen-sensitive mapping using dual-energy computed tomography. Skeletal Radiol 2021;50(7):1359–1367. <https://doi.org/10.1007/s00256-020-03685-5>

**Shared subjects: n = 8**

**4.** Stelbrink C, Jahnke P, Goehler F, et al. Diagnostic accuracy of dual energy computed tomography for suspected pyogenic spondylodiscitis. Sci Rep 2025;15:20040. <https://doi.org/10.1038/s41598-025-04216-9>

**Shared subjects: n = 12**
