# Supplementary material for: Quantification of intervertebral disc degeneration using dual-energy computed tomography collagen maps: a comparative study with MRI Pfirrmann grading
Source: Sci Rep. 2026 Sep 3;16:27687. doi: 10.1038/s41598-026-69070-9 (PMC13542269; doi:10.1038/s41598-026-69070-9)
Supplement: Supplementary file 2 [file 41598_2026_69070_MOESM11_ESM.docx]

Supplementary Figure 1(A/B): Boxplot of DECT collagen and chondroitin densities (HU) in AF and NP in female (A) and male (B) patients.

In female patients, AF and NP density constantly decrease with increasing PF-grades. AF ad NP densities appear to be more variable in male patients.

AF = anulus fibrosus, NP = nucleus pulposus, DECT= dual-energy computed tomography

Supplementary Figure 2(A/B): Boxplots of mean IVD density (HU) in DECT cMaps (A) and in DECT 135-kVp images.

A constant reduction of IVD density with increasing PF-grade is apparent in cMaps (A) but not in 135-kVp DECT images (B).

AF = anulus fibrosus, NP = nucleus pulposus, DECT = dual-energy computed tomography

Supplementary Figure 3: Bar chart of PF-grade distribution in percent separated by IVD Level.

Barchart showing that PF-grade II is most frequent at all IVD levels investigated in our patient population. PF-grades I and V are least frequent.

*Supplementary Figure 4*: Example of ROI placement in cMap and MRI images. We placed 3 ROI´s in each IVD in both cMap and MRI, additionally one in the CSF in MRI images.

*ROI= Region of interest, IVD= intervertebral disc*

Supplementary Table 1: Linear mixed model assessing change in AF and NP in 135-kVp DECT images.

Comparing male and female, AF density in male is slightly elevated by 0.21 HU. NP density in male is lower by -0.79 HU. Looking at AF density, no statically significant difference in-between PF-grades is noticeable, but a trend of density reduction is visible. NP density in PF-grade IV shows a significant difference compared to PF-grade I (mean difference PF-grade IV = 9.44 HU, p = 0.048).

AF = anulus fibrosus; NP = nucleus pulposus; DECT = dual-energy computed tomography; Ref. = reference level for comparison

Supplementary Table 2: Linear mixed model assessing change in AF and NP in MRI SI.

Comparing males and females, a significant difference in AF and NP SI is visible. Analysis shows a statistically significant difference in AF in discs graded as PF-grade III to V compared to PF-grade I. A significant difference in NP SI values between discs graded as PF-grade II to V compared to PF-grade I is observed.

AF = anulus fibrosus; NP = nucleus pulposus; MRI = magnetic resonance imaging; SI = signal intensity; Ref. = reference level for comparison.

Supplementary Table 3: Linear mixed model estimating mean AF and NP density in 135-kVp DECT images and MRI separated by PF-grade.

AF and NP densities in 135-kVp DECT images show no major difference in-between PF-grades. Regarding MRI SI a reduction from PF-grade I to V is visible in AF as well as in NP. Estimates for male and female densities and SI´s stay similar.

AF = anulus fibrosus; NP = nucleus pulposus; DECT = dual-energy computed tomography

Supplementary Table 4: Summary of mean IVD collagen and chondroitin densities in DECT cMap by IVD level for males and females.

DECT= dual-energy computed tomography; IVD = intervertebral disc; SD = standard deviation

*Supplementary Table 5:* Descriptive table comparing mean IVD density in males and females in DECT cMaps, conventional CT and MRI SI separated by PF-grade.

Table showing a visible disc density reduction in DECT cMap within female and male patients with increasing PF-grade. The lowest disc density is observed in PF-grade V. Density between male and female patients differ in-between PF-grades. In conventional CT disc density scatters around 100 HU in different PF-grades. Total mean disc density is lowest at PF-grade V. MRI SI reduces from PF-grade I to V, with V showing the lowest SI.

DECT= dual-energy computed tomography; MRI = magnetic resonance imaging; SD = standard deviation; SI = signal intensity

*Supplementary document 6:* Patient overlap with earlier departmental studies

Overlap of the study population with four earlier investigations from our department that drew on the same routinely acquired dual-energy CT examinations. The document lists each source study, its research question and the number of shared subjects.
