# Supplementary material for: Quantification of intervertebral disc degeneration using dual-energy computed tomography collagen maps: a comparative study with MRI Pfirrmann grading
Source: Sci Rep. 2026 Sep 3;16:27687. doi: 10.1038/s41598-026-69070-9 (PMC13542269; doi:10.1038/s41598-026-69070-9)
Supplement: Supplementary file 3 [file 41598_2026_69070_MOESM5_ESM.docx]

Supplementary Table 1: Linear mixed model assessing change in AF and NP in 135-kVp DECT.

| Estimates of Fixed Effects 135-kVp AF | | | | |
| --- | --- | --- | --- | --- |
| Parameter | Estimate | Sig. | 95% Confidence Interval | |
|  |  |  | Lower Bound | Upper Bound |
| Male | 0.21 | 0.889 | -4.19 | 4.60 |
| Female | ref. | ref. | ref. | ref. |
| PF-grade I | ref. | ref. | ref. | ref. |
| PF-grade II | -0.29 | 0.950 | -9.47 | 8.88 |
| PF-grade III | -4.37 | 0.363 | -13.84 | 5.10 |
| PF-grade IV | -1.39 | 0.782 | -11.25 | 8.48 |
| PF-grade V | -6.36 | 0.407 | -21.45 | 8.73 |
| Estimates of Fixed Effects 135-kVp NP | | | | |
| Male | -0.79 | 0.791 | -5.12 | 3.55 |
| Female | ref. | ref. | ref. | ref. |
| PF-grade I | ref. | ref. | ref. | ref. |
| PF-grade II | 3.85 | 0.380 | -4.78 | 12.49 |
| PF-grade III | 3.55 | 0.434 | -5.40 | 12.51 |
| PF-grade IV | 9.44 | 0.048 | 0.09 | 18.79 |
| PF-grade V | 0.74 | 0.917 | -13.24 | 14.72 |
