# Supplementary material for: Quantification of intervertebral disc degeneration using dual-energy computed tomography collagen maps: a comparative study with MRI Pfirrmann grading
Source: Sci Rep. 2026 Sep 3;16:27687. doi: 10.1038/s41598-026-69070-9 (PMC13542269; doi:10.1038/s41598-026-69070-9)
Supplement: Supplementary file 4 [file 41598_2026_69070_MOESM6_ESM.docx]

Supplementary Table 2: Linear mixed model assessing change in AF and NP in MRI SI.

| Estimates of Fixed Effects MRI SI AF | | | | |
| --- | --- | --- | --- | --- |
| Parameter | Estimate | Sig. | 95% Confidence Interval | |
|  |  |  | Lower Bound | Upper Bound |
| Male | 0.04 | 0.025 | 0.005 | 0.075 |
| Female | ref. | ref. | ref. | ref. |
| PF-grade I | ref. | ref. | ref. | ref. |
| PF-grade II | 0.001 | 0.969 | -0.05 | 0.05 |
| PF-grade III | -0.08 | 0.003 | -0.14 | -0.03 |
| PF-grade IV | -0.16 | <0.001 | -0.22 | -0.10 |
| PF-grade V | -0.18 | <0.001 | -0.25 | -0.09 |
| Estimates of Fixed Effects MRI SI NP | | | | |
| Male | 0.074 | 0.008 | 0.020 | 0.127 |
| Female | ref. | ref. | ref. | ref. |
| PF-grade I | ref. | ref. | ref. | ref. |
| PF-grade II | -0.11 | 0.013 | -0.19 | -0.02 |
| PF-grade III | -0.36 | <0.001 | -0.45 | -0.27 |
| PF-grade IV | -0.54 | <0.001 | -0.63 | -0.44 |
| PF-grade V | -0.52 | <0.001 | -0.66 | -0.39 |
