# Supplementary material for: Quantification of intervertebral disc degeneration using dual-energy computed tomography collagen maps: a comparative study with MRI Pfirrmann grading
Source: Sci Rep. 2026 Sep 3;16:27687. doi: 10.1038/s41598-026-69070-9 (PMC13542269; doi:10.1038/s41598-026-69070-9)
Supplement: Supplementary file 5 [file 41598_2026_69070_MOESM7_ESM.docx]

Supplementary Table 3: Linear mixed model estimating mean AF and NP density in 135kVp DECT and MRI separated by PF-grade.

| Estimates PF-grade 135-kVp AF | | | | Estimates PF-grade MRI AF | | | |
| --- | --- | --- | --- | --- | --- | --- | --- |
| PF-grade | Mean | 95% CI | | PF-grade | Mean | 95% CI | |
|  |  | Lower Bound | Upper Bound |  |  | Lower Bound | Upper Bound |
| I | 104.92 | 95.99 | 113.86 | I | 0.31 | 0.26 | 0.37 |
| II | 104.63 | 101.48 | 107.78 | II | 0.32 | 0.29 | 0.33 |
| III | 100.55 | 97.27 | 103.83 | III | 0.23 | 0.21 | 0.25 |
| IV | 103.54 | 99.30 | 107.77 | IV | 0.15 | 0.13 | 0.18 |
| V | 98.57 | 86.41 | 110.72 | V | 0.14 | 0.07 | 0.20 |
| Male | 102.5 | 98.4 | 106.7 | Male | 0.25 | 0.22 | 0.28 |
| Female | 102.3 | 98.4 | 106.3 | Female | 0.21 | 0.18 | 0.24 |
| Estimates PF-grade 135-kVp NP | | | | Estimates PF-grade MRI NP | | | |
| I | 85.58 | 77.09 | 94.07 | I | 0.75 | 0.66 | 0.83 |
| II | 89.43 | 86.40 | 92.47 | II | 0.64 | 0.60 | 0.67 |
| III | 89.14 | 86.04 | 92.23 | III | 0.39 | 0.36 | 0.42 |
| IV | 95.02 | 91.02 | 99.01 | IV | 0.21 | 0.17 | 0.25 |
| V | 86.32 | 75.22 | 97.42 | V | 0.22 | 0.12 | 0.33 |
| Male | 88.71 | 84.71 | 92.70 | Male | 0.48 | 0.43 | 0.52 |
| Female | 89.49 | 85.71 | 93.27 | Female | 0.40 | 0.36 | 0.45 |
