# Supplementary material for: Quantification of intervertebral disc degeneration using dual-energy computed tomography collagen maps: a comparative study with MRI Pfirrmann grading
Source: Sci Rep. 2026 Sep 3;16:27687. doi: 10.1038/s41598-026-69070-9 (PMC13542269; doi:10.1038/s41598-026-69070-9)
Supplement: Supplementary file 6 [file 41598_2026_69070_MOESM8_ESM.docx]

Supplementary Table 4: Summary of mean IVD collagen and chondroitin densities in DECT cMap by IVD level for males and females.

|  | IVD Level |  | N | SD |
| --- | --- | --- | --- | --- |
| Male | T12-L1 | 124.91 | 26 | 25.80 |
|  | L1-L2 | 115.10 | 26 | 24.21 |
|  | L2-L3 | 109.28 | 29 | 25.33 |
|  | L3-l4 | 114.18 | 27 | 23.46 |
|  | L4-L5 | 114.32 | 26 | 23.01 |
|  | L5-S1 | 134.16 | 26 | 33.98 |
|  | Total | 118.46 | 160 | 27.11 |
| Female | T12-L1 | 118.97 | 25 | 29.96 |
|  | L1-L2 | 114.20 | 28 | 32.25 |
|  | L2-L3 | 108.32 | 34 | 24.82 |
|  | L3-l4 | 109.90 | 34 | 25.93 |
|  | L4-L5 | 105.38 | 27 | 24.10 |
|  | L5-S1 | 121.36 | 31 | 40.93 |
|  | Total | 112.84 | 179 | 30.28 |
