# Supplementary material for: Quantification of intervertebral disc degeneration using dual-energy computed tomography collagen maps: a comparative study with MRI Pfirrmann grading
Source: Sci Rep. 2026 Sep 3;16:27687. doi: 10.1038/s41598-026-69070-9 (PMC13542269; doi:10.1038/s41598-026-69070-9)
Supplement: Supplementary file 7 [file 41598_2026_69070_MOESM9_ESM.docx]

*Supplementary Table 5:* Descriptive table comparing mean IVD density in males and females in DECT cMaps, conventional CT and MRI SI separated by PF-grade.

| **PF-grade** | | **cMap (HU)** | | | **CT (HU)** | | | **MRI (SI)** | | |
| --- | --- | --- | --- | --- | --- | --- | --- | --- | --- | --- |
|  |  | Total | Male | Female | Total | Male | Female | Total | Male | Female |
| **I** | Mean | 122.95 | 117.65 | 128.26 | 97.86 | 93.54 | 102.18 | 0.53 | 0.55 | 0.51 |
|  | N | 18 | 9 | 9 | 18 | 9 | 9 | 18 | 9 | 9 |
|  | SD | 27.15 | 30.31 | 24.17 | 10,68 | 7.57 | 11.96 | 0.07 | 0.08 | 0.05 |
|  | Min | 83.70 | 83.70 | 103.76 | 85.31 | 85.31 | 89.65 | 0.42 | 0.43 | 0.42 |
|  | Max | 184.62 | 184.62 | 169.38 | 126.80 | 104.22 | 126.80 | 0.69 | 0.69 | 0.58 |
| **II** | Mean | 121.29 | 122.15 | 120.15 | 101.76 | 102.63 | 100.61 | 0.45 | 0.48 | 0.41 |
|  | N | 146 | 83 | 63 | 146 | 83 | 63 | 146 | 83 | 63 |
|  | SD | 27.13 | 23.29 | 31.66 | 14.35 | 15.85 | 12.13 | 0.12 | 0.12 | 0.12 |
|  | Min | 25.75 | 62.39 | 25.75 | 74.87 | 80.29 | 74.87 | 0.14 | 0.21 | 0.14 |
|  | Max | 231.62 | 186.47 | 231.62 | 185.97 | 185.97 | 141.51 | 0.75 | 0.75 | 0.62 |
| **III** | Mean | 112.92 | 120.48 | 105.92 | 96.62 | 97.69 | 95.63 | 0.29 | 0.31 | 0.27 |
|  | N | 102 | 49 | 53 | 102 | 49 | 53 | 102 | 49 | 53 |
|  | SD | 30.27 | 29.18 | 29.83 | 9.99 | 9.72 | 10.23 | 0.11 | 0.09 | 0.12 |
|  | Min | 37.49 | 51.52 | 37.49 | 75.00 | 77.11 | 75.00 | 0.10 | 0.10 | 0.10 |
|  | Max | 207.57 | 207.57 | 171.69 | 140.19 | 140.19 | 117.68 | 0.56 | 0.48 | 0.56 |
| **IV** | Mean | 107.31 | 98.29 | 110.13 | 97.89 | 91.04 | 100.04 | 0.14 | 0.17 | 0.13 |
|  | N | 67 | 16 | 51 | 67 | 16 | 51 | 67 | 16 | 51 |
|  | SD | 27.70 | 27.03 | 27.56 | 14.00 | 8.34 | 14.77 | 0.06 | 0.07 | 0.06 |
|  | Min | 50.87 | 50.87 | 52.06 | 74.63 | 80.42 | 74.63 | 0.05 | 0.08 | 0.05 |
|  | Max | 161.19 | 157.05 | 161.19 | 154.49 | 104.24 | 154.49 | 0.39 | 0.33 | 0.39 |
| **V** | Mean | 87.27 | 93.01 | 81.54 | 94.53 | 96.35 | 92.71 | 0.14 | 0.13 | 0.15 |
|  | N | 6 | 3 | 3 | 6 | 3 | 3 | 6 | 3 | 3 |
|  | SD | 29.73 | 41.24 | 20.27 | 34.81 | 52.06 | 17.55 | 0.03 | 0.04 | 0.03 |
|  | Min | 45.50 | 45.50 | 63.03 | 37.77 | 37.77 | 75.86 | 0.08 | 0.08 | 0.11 |
|  | Max | 199.54 | 119.54 | 103.20 | 137.32 | 137.32 | 110.89 | 0.17 | 0.17 | 0.16 |
